# Prevalence of depression and anxiety in patients with Tourette syndrome; 1997 to 2022: a systematic review and meta-analysis

**DOI:** 10.1186/s13052-023-01562-0

**Published:** 2023-12-01

**Authors:** Parvin Abbasi, Sepideh Tanhaie, Mohsen Kazeminia

**Affiliations:** 1 Department of Nursing, School of Nursing and Midwifery, Kermanshah University of Medical Sciences, Kermanshah, Iran; 2 School of Medicine, Kermanshah University of Medical Sciences, Kermanshah, Iran; 3 Student Research Committee, Kermanshah University of Medical Sciences, Kermanshah, Iran

**Keywords:** Prevalence, Depression, Anxiety, Tourette syndrome, Meta-analysis

## Abstract

Tourette Syndrome (TS) is a disorder in which the patient has a history of multiple motor and vocal tics. Depression and anxiety are common in these patients. The results of the studies show different prevalence of these disorders in patients with TS. So, the objective of the present study was to liken the prevalence of depression and anxiety in patients with TS by systematic review and meta-analysis. The present study was conducted according to PRISMA guidelines during 1997–2022. The articles were obtained from Scopus, Embase, PubMed, Web of Science (WoS) and Google Scholar databases. I^2^ was used to investigate heterogeneity between studies. Data were analyzed by comprehensive meta-analysis software (Version 2). Finally, 12 articles with a sample size of *n* = 3812 were included in the study. As a result of combining the results of the studies, the total estimate of the prevalence of depression and anxiety in patients with TS was 36.4% (95% confidence interval: 21.1–54.9%) and 53.5% (95% confidence interval: 39.9–66.6%), respectively. The results of meta-regression showed that by increasing mean age (9–31.5 years), the prevalence of depression and anxiety in patients with TS increased significantly (*P*<0.001). The results of the present study showed that the prevalence of depression and anxiety was high in patients with TS. Therefore, it is suggested that health officials and policy makers design measures to prevent and control these disorders.

## Introduction

Disorders of early life remaining throughout life are called neurodevelopmental disorders [1]. These disorders usually appear early in a child's development, often before they reach school age. Defects associated with these disorders include personal, social, educational, or occupational dysfunction. These disorders may show significant changes over time [1, 2]. Childhood and adolescence disorders can delay and / or even hinder children’s social development [3].

Tic ​​disorders are a group of neurodevelopmental disorders that commonly begin in childhood and adolescence and may be constant or alternatively severe over time [1, 3]. Tics are repetitive, involuntary, inharmonic and sudden movements or sounds that can involve distinct muscle groups and often appear between the ages of 4 and 6. These disorders are divided into TS, chronic motor or vocal tics, and transient tics [4]. The prevalence of tic disorders is higher in children than adults. Thus, about 5–30 out of ten thousand children and only 1–2 out of ten thousand adults have this disorder [5].

Diagnostic criteria for Tourette syndrome based on DSM-5 include (1) multiple motor tics and one or more vocal tics sometimes present during the disease (though not necessarily simultaneously), (2) the frequency of tics may increase and reduce, but continue for more than a year since the beginning of the first tick, (3) before 18 years old, (4) this disorder is not caused by the effects of drugs (such as cocaine) or other physical diseases such as Huntington's disease, inflammation of the brain after the virus, and etc. [4]. The meta-analysis of TS in China reported the prevalence in children as 1.7% [6], 0.52% in USA [7] and 0.11% in Polish in adults [8]. The prevalence of TS in boys is 3 times higher than in girls [5].

Anxiety and depression are among the most important issues studied by psychologists, psychiatrists and behavioural scientists around the world [9]. Among physical and mental diseases, depression is the number one problem in the world. Depression is one of the most important mood disorders that is associated with low mood, loss of interest, guilt and worthlessness, sleep and appetite disorders, reduced energy and poor concentration. Depression and anxiety are the most common psychiatric disorders with a prevalence of 10–20% in the general population [10]. Approximately 15% of the total population experience a period of major depression at some point in their lives [11]. Anxiety is an unpleasant and unknown state that affects a person and is accompanied by symptoms such as fatigue, restless and heartbeat. The genetic, hereditary, environmental, psychological, social and biological factors are involved in the etiology of anxiety [12]. A person who is constantly exposed to anxiety loses his self-confidence and feels depressed while feeling humiliated, which in turn will fuel the vicious cycle of job stress and efficiency. Continuation of this cycle can gradually erode the mental and physical abilities of individuals and after a while lead to unstable nervous disorders [13].

Several preliminary studies have been conducted on the prevalence of depression and anxiety in patients with TS in different parts of the world, but these studies have investigated the prevalence in a small environment with a smaller sample size. The results of studies showed different prevalence of these disorders in different populations. Also, none of these studies have investigated the effect of potential factors such as age and prevalence over time. So, the objective of the present study was to standardize the prevalence of depression and anxiety in patients with TS by systematic review and meta-analysis.

## Methods

The present systematic review and meta-analysis was conducted according to PRISMA 2020 guidelines [14] during 1997–2022. The articles entered the meta-analysis were obtained from Scopus, Embase, PubMed, Web of Science (WoS) and Google Scholar databases. Keywords used in the search included "Prevalence", "Epidemiology", "Prevalent*", "Tourette syndrome", "Depression", "Depressive "Disorder", "Depress*", "Anxiety*", and "Anxieties" and in combination using (or) and (and) operators. Keywords were validated using MeSh for PubMed and Emtree for Embase. The study search did not consider any time or language restrictions to retrieve all possible related articles by January 2022. Finally, Google Scholar and the references of all articles entered the meta-analysis were manually searched. For example, PubMed search strategy was defined as follows:

(((((((((Epidemiology[MeSH Terms]) OR (Prevalence[MeSH Terms])) OR (Epidemiology[Title/Abstract])) OR (Prevalance[Title/Abstract])) OR (Prevalence[Title/Abstract])) OR (Prevalent*[Title/Abstract])) OR ("Prevalences"[Title/Abstract])) OR ("Prevalence s"[Title/Abstract])) AND ((("Tourette's syndrome"[Title/Abstract]) OR ("Tourette syndrome"[Title/Abstract])) OR ("Tourette syndrome"[MeSH Terms]))) AND ((((Depression[MeSH Terms]) OR ("Depressive Disorder"[MeSH Terms])) OR (Depress*[Title/Abstract])) OR (((Anxiety[MeSH Terms]) OR (Anxiety*[Title/Abstract])) OR (Anxieties[Title/Abstract]))).

In order to reduce publication bias and error, all stages of searching in different databases, review, selection, data extraction and quality evaluation of articles were performed by two researchers, and in case of disagreement, first with discussion, then review and finally according to the opinion of the third person, an agreement was reached.

### Inclusion criteria

1. Original Research Articles
2. Observational articles (cross-sectional study, case study and cohort study)
3. Access to the full text of the article
4. Studies that reported the percentage or frequency of prevalence of depression or anxiety in patients with TS.

### Exclusion criteria

1. Studies unrelated to the objective of the study
2. Interventional studies (clinical trial study, field trial study and social trial study), qualitative studies, case series, case reports, letter to editor, articles presented at conferences, reviews, systematic review and meta-analysis, dissertations and animal studies
3. The full text of the article is not available
4. Repeated and overlapping studies in different databases

### Selection process of studies

After determining the search strategy for each database, all articles obtained from different databases were entered EndNote X8 software. First, all repeated and overlapping studies in different databases were excluded. The names of the authors, institutes and journals of all studies were then excluded. At the next stage, the title and abstract of the studies were reviewed and unrelated studies were excluded. Then, full text of the remaining articles were thoroughly reviewed according to inclusion and exclusion criteria and irrelevant studies were excluded. Finally, articles that met all inclusion criteria entered the qualitative evaluation.

### Qualitative evaluation of studies

Qualitative evaluation of studies was performed using Joanna Briggs Institute (JBI) checklist, which is a standard and well-known checklist for qualitative evaluation of prevalence studies [15]. This checklist has 9 different questions about: (1) sample frame, (2) participants, (3) sample size, (4) study subject and setting described in detail), (5) data analysis, (6) valid methods for identifying conditions, (7) measure the situation, (8) statistical analysis and (9) adequate response rate. For scoring, "Yes" was awarded if mentioned, "No" was awarded if not mentioned, and "NA" was awarded if not reported. The minimum and maximum scores based on the number of "Yes" were 0 and 9, respectively. The results of qualitative evaluation of studies based on JBI checklist items are reported in Table 1.

**Table 1:** Qualitative evaluation of studies based on JBI checklist items

First author, year (reference)	Sample frame	Participants	Sample size	Study subjects	Data analysis	Methods	Measure the situation	Statistical analysis	Response rate adequate	Quality score (Number “Yes”)
BSc, 1998 [16]	Yes	Yes	Yes	No	Yes	NA	NA	Yes	No	5
Robertson, 2006 [17]	Yes	Yes	Yes	No	Yes	NA	NA	Yes	No	5
Berthier, 1998 [18]	No	Yes	Yes	No	Yes	Yes	Yes	Yes	No	6
Robertson, 1997 [19]	No	NA	Yes	No	Yes	Yes	Yes	Yes	No	5
Gharatya, 2014 [20]	Yes	NA	Yes	No	Yes	Yes	Yes	Yes	No	6
Whitney, 2019 [21]	No	NA	Yes	Yes	Yes	Yes	NA	Yes	No	5
Robertson, 2015 [22]	Yes	Yes	Yes	No	Yes	Yes	Yes	Yes	No	7
Rizzo, 2017 [23]	No	Yes	Yes	Yes	Yes	Yes	Yes	Yes	Yes	8
Robertson, 2002 [24]	Yes	Yes	Yes	No	Yes	NA	NA	Yes	No	5
Baglioni, 2014 [25]	Yes	Yes	Yes	No	Yes	NA	NA	Yes	Yes	6
Solís-García, 2021 [26]	Yes	Yes	Yes	No	Yes	NA	Yes	Yes	Yes	7
Wodrich, 1997 [27]	No	NA	Yes	Yes	Yes	Yes	Yes	Yes	No	6

### Data extraction

Data were extracted using a pre-prepared checklist. The various items on this checklist included name of the corresponding author, year of publication of the article, sample size (total, male and female), country, diagnostic tools for depression and anxiety, prevalence, age of patients and population studied.

### Statistical analysis

The index studied in this study was the prevalence of depression and anxiety in patients with TS, which was used to combine the results of different studies on the frequency in each study. Heterogeneity between studies was investigated by I^2^ and due to the high heterogeneity between the results of studies included in the meta-analysis (I^2^˃ 75%), the random effects model was used. Funnel Plot and Egger’s regression intercept were used to investigate publication bias. Meta-regression was also used to investigate the relationship between the prevalence of depression and anxiety in patients with TS with sample size, year of publication and mean age of patients. Subgroup analysis was performed according to the study population. Data analysis was performed by Comprehensive Meta-Analysis software (Version 2) and P value less than 0.05 was considered as statistically significant.

## Results

### Stages of articles entry in meta-analysis

**Fig. 1:**
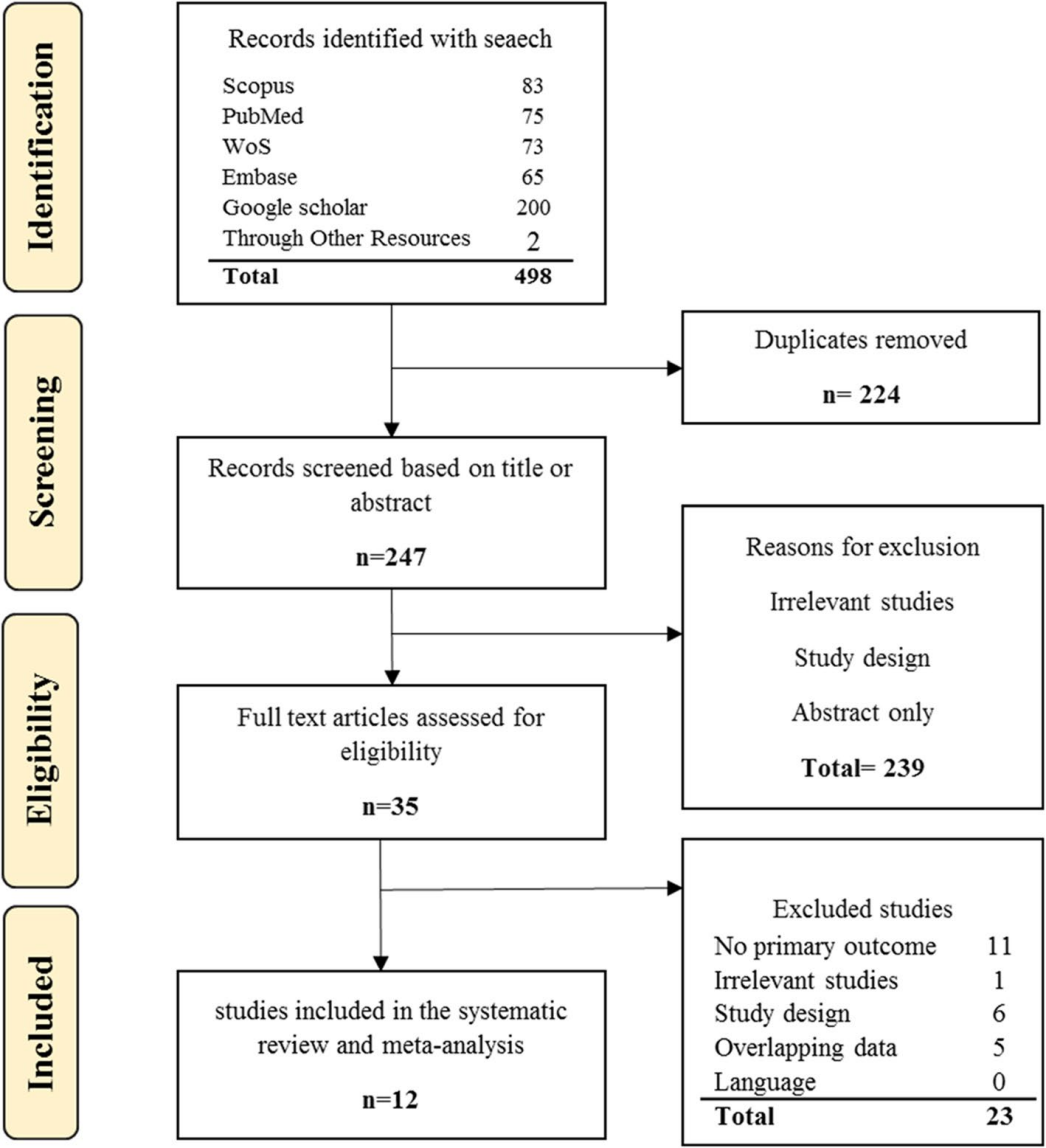
PRISMA 2020 flow diagram for article selection

A total of 496 studies were found in the initial search using the search strategies identified for the various databases. 2 studies were added through manual search. 224 studies were repeated in different databases and excluded. 274 studies were reviewed by title and abstract, of which 239 studies were excluded due to irrelevance. Full-text of remaining 35 studies was reviewed, of which 23 studies were excluded due to not meeting all inclusion criteria. Finally, the remaining 12 articles entered the qualitative evaluation and none of the studies based on JBI checklist were of poor quality. The stages of PRISMA 2020 flow chart are shown in Fig. 1.

### General information of the articles

The total sample size of the studies was *n* = 3812. The highest and lowest sample sizes were related to the 2019 study in the USA [21] with *n* = 1428 and the 2021 study in Spain [26] with *n* = 22, respectively. Two-thirds of the studies have been conducted on children or adolescents. The oldest and newest studies were in 1997 and 2021, respectively. 50% of the studies have been conducted in the UK. Also, the studied studies reported the prevalence of depression between 8.7% and 73% and the prevalence of anxiety between 31.4% and 80%. The data of the articles entered systematic review and meta-analysis are given in Table 2.

**Table 2:** Specifications and data of articles entered for systematic review and meta-analysis

**First author, year, (reference)**	**Country**	**Sample size (n)**			**Age (year)**	**Diagnostic tool**		**Prevalence (%)**		**Population**
		**Total**	**Male**	**Female**		**Depression**	**Anxiety**	**Depression**	**Anxiety**	
BSc, 1998 [16]	UK	30	17	13	9.0	BCDI	-	29.0	-	Children
Robertson, 2006 [17]	UK	918	-	-	9.0	DSM-IV-TR	-	13.0	-	Children
Berthier, 1998 [18]	Spain	30	13	17	31.5 ± 11.0	HDRS	TBSA	53.0	73.0	Adult
Robertson, 1997 [19]	UK	39	31	8	26.2 (11–55)	BDI	Spielberger	12.3	44.6	Adult
Gharatya, 2014 [20]	UK	524	-	-	26.9	BDI	Spielberger	75.0	80.0	Adult
Whitney, 2019 [21]	USA	1428	-	-	11.5	BDI	Spielberger	15.3	37.9	Children and Adolescents
Robertson, 2015 [22]	UK	578	422	156	25.4 ± 14.3	DSM	DSM	49.0	43.0	Adult
Rizzo, 2017 [23]	Italy	98	81	17	12.2 ± 0.7	CDI	MASC	66.7	44.8	Children and Adolescents
Robertson, 2002 [24]	UK	57	45	12	10.7 ± 3.0	DSM-III-R	-	8.7	-	Children and Adolescents
Baglioni, 2014 [25]	Italy	55	40	15	17.6	DSM-IV-TR	DSM-IV-TR	27.44	31.4	Children and Adolescents
Solís-García, 2021 [26]	Spain	22	19	3	11.0	DSM-5	DSM-5	50.0	72.7	Children and Adolescents
Wodrich, 1997 [27]	USA	33	25	8	9.4 ± 2.3	DSM-III-R	DSM-III-R	73.0	55.0	Children and Adolescents

### Meta-analysis of the prevalence of depression and anxiety in patients with TS

**Fig. 2:**
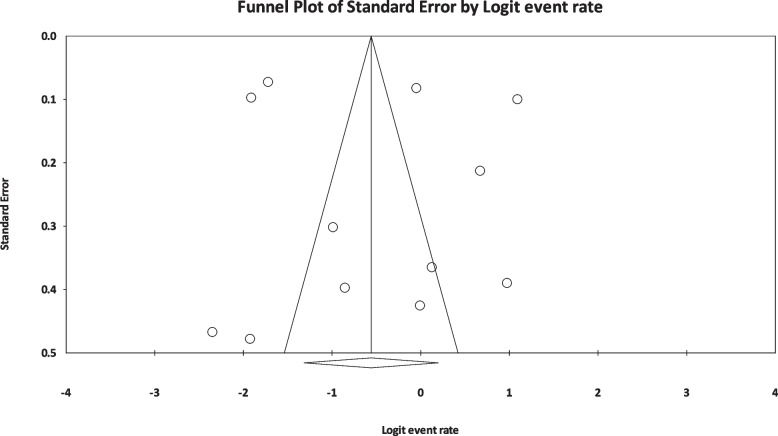
Results of funnel plot for estimating the total prevalence of depression

**Fig. 3:**
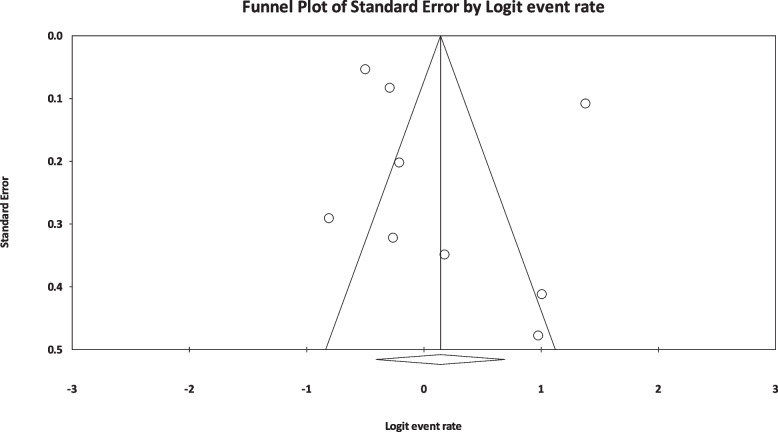
Results of funnel plot for estimating the total prevalence of anxiety

**Fig. 4:**
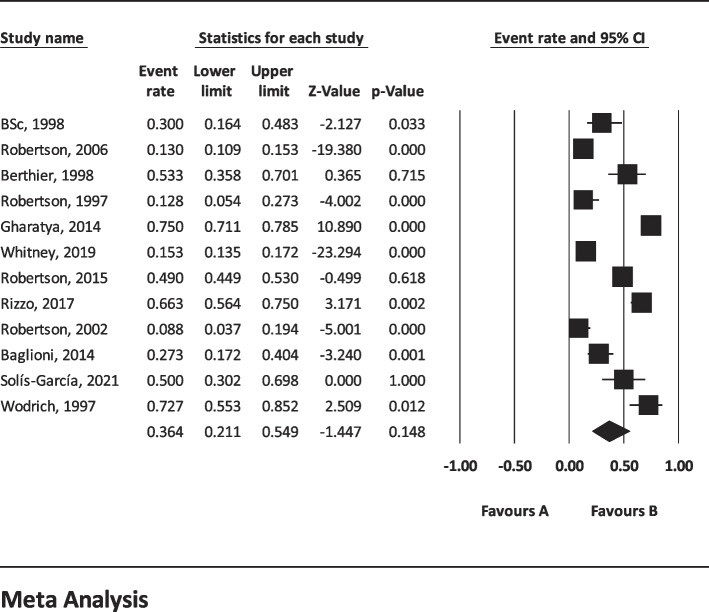
Forest plot for estimating the total prevalence of depression

**Fig. 5:**
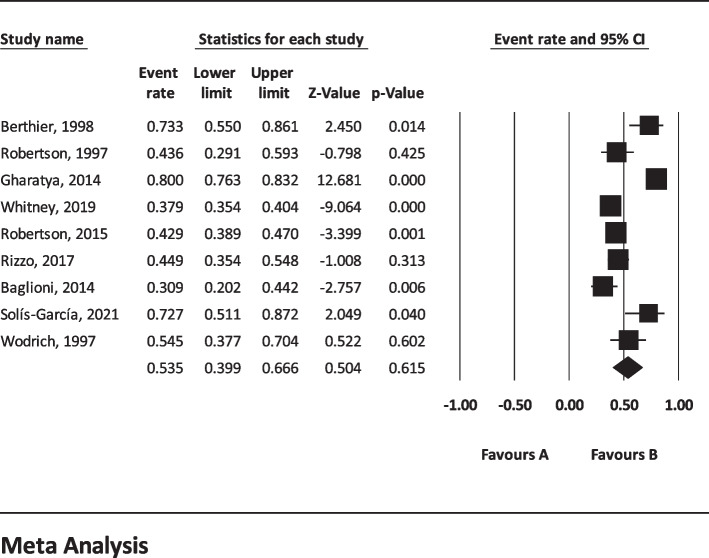
Forest plot for estimating the total prevalence of anxiety

**Fig. 6:**
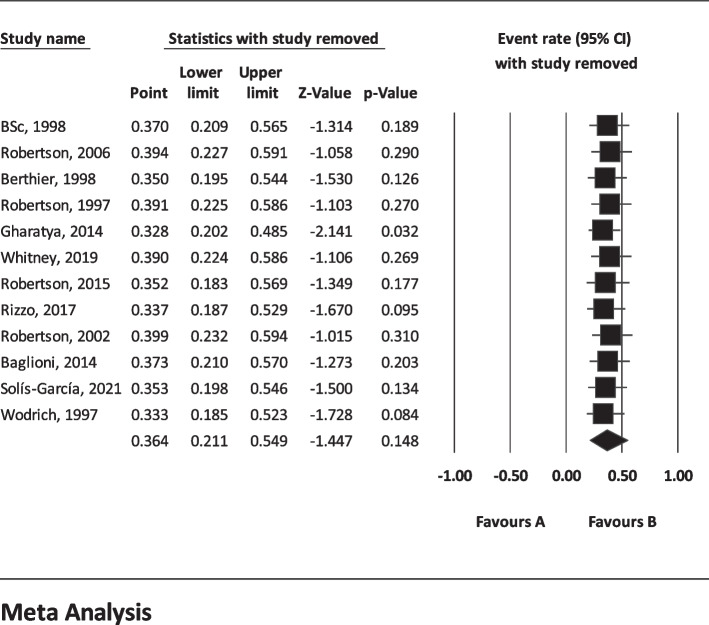
Sensitivity analysis for estimating the total prevalence of depression

**Fig. 7:**
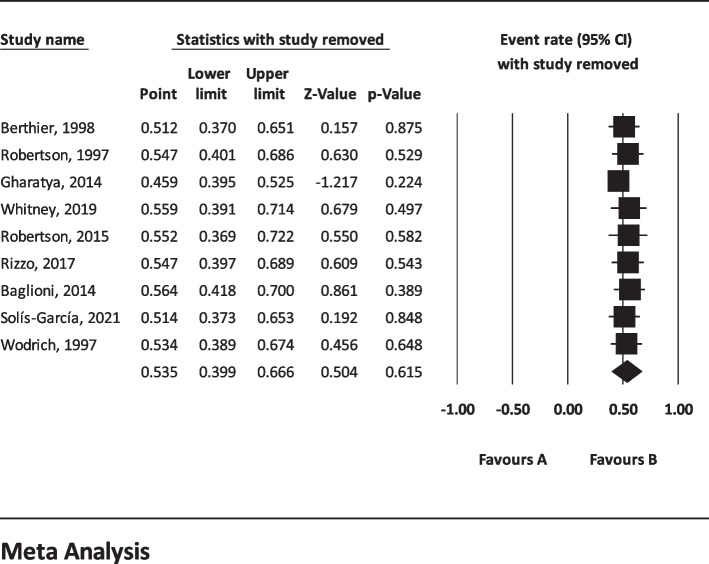
Sensitivity analysis chart for estimating the total prevalence of anxiety

The results of I^2^ test for the global prevalence of depression and anxiety in patients with TS indicated a significant heterogeneity between studies (depression = 98.63 and anxiety = 96.91). So, random effect model was used for data analysis (Table 3). According to funnel plot (Figs. 2 and 3) and the results of Egger’s regression intercept, there was no publication bias among the studies at the level of 0.1 (depression = 0.685 and anxiety = 0.410). As a result of combining the results of all studies, the prevalence of depression in patients with TS; 36.4% (95% confidence interval: 21.1–54.9%) and prevalence of anxiety in patients with TS; 53.5% (95% confidence interval: 39.9–66.6%) was estimated by random effect model (Figs. 4 and 5) (black square percentage and the length of the line on which the 95% confidence interval is located in each study, the rhomb represents the total estimate of the prevalence). The results of sensitivity analysis showed that by excluding each of the studies, the final estimate of the prevalence percentage does not change significantly (Figs. 6 and 7).

**Table 3:** Report the results of fixed and random effects model on meta-analysis

Disorder	Model	Number studies	Point estimate	Lower limit	Upper limit	Z-value	*P*-value	Q-value	df (Q)	*P*-value	I^2^	Tau squared	Standard error	Variance	Tau
Depression	Fixed	12	0.330	0.312	0.348	-17.36	0.000	808.173	11	0.000	98.639	1.692	1.189	1.414	1.301
	Random	12	0.364	0.211	0.549	-1.44	0.148								
Anxiety	Fixed	9	0.462	0.442	0.481	-3.821	0.000	259.627	8	0.000	96.919	0.629	0.547	0.299	0.793
	Random	9	0.535	0.399	0.666	0.504	0.615

### Meta-regression of prevalence of depression and anxiety in patients with TS

**Fig. 8:**
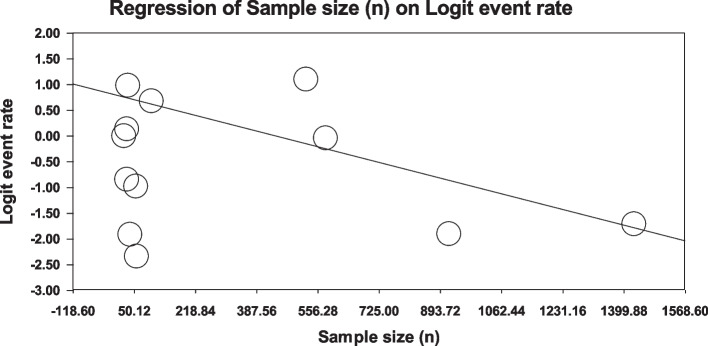
Meta-regression of the relationship between sample size and prevalence depression

**Fig. 9:**
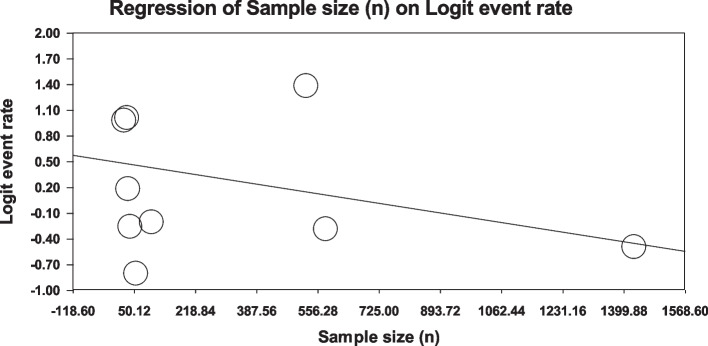
Meta-regression of the relationship between sample size and prevalence anxiety

**Fig. 10:**
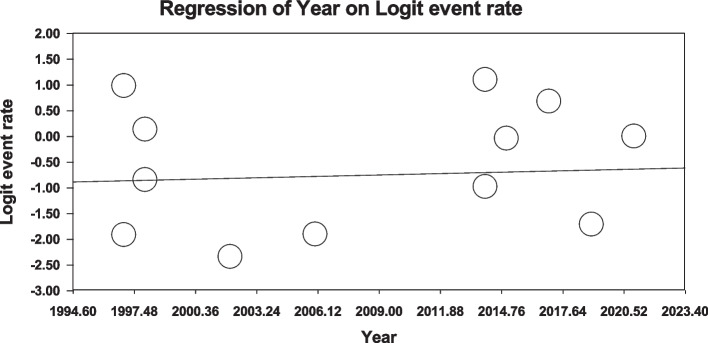
Meta-regression between years of study and prevalence depression

**Fig. 11:**
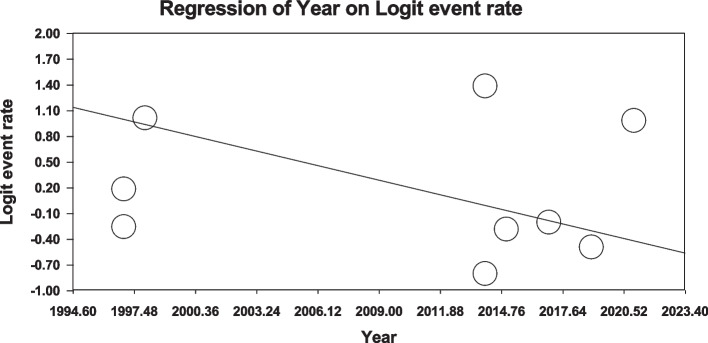
Meta-regression between years of study and prevalence anxiety

**Fig. 12:**
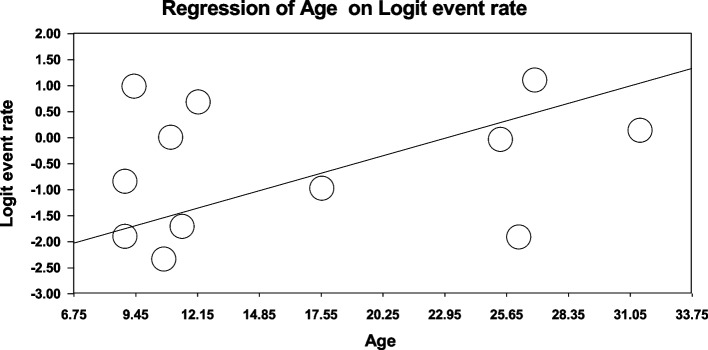
Meta-regression between the mean age of patients and prevalence depression

**Fig. 13:**
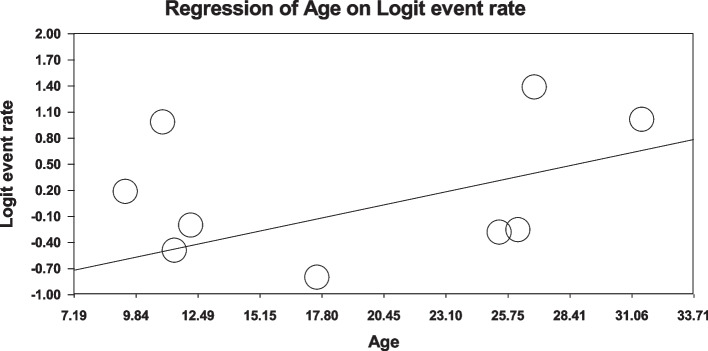
Meta-regression of the relationship between the mean age of patients and prevalence anxiety

Using meta-regression, the relationship between potential factors such as sample size (Figs. 8 and 9), year of study (Figs. 10 and 11) and mean age of patients (Figs. 12 and 13) and the total estimate of the prevalence of depression and anxiety in patients with TS was investigated. The results showed that by increasing sample size, the prevalence of depression (Fig. 8) and anxiety (Fig. 9) reduced significantly (*P*<0.001). By increasing year of study, the prevalence of anxiety (Fig. 11) reduced significantly (*P*<0.001), but the relationship between year of study and prevalence of depression (Fig. 10) was not significant (*P*˃0.05). By increasing mean age (9–31.5 years) of patients, the prevalence of depression (Fig. 12) and anxiety (Fig. 13) increased significantly (*P*<0.001).

### Subgroup analysis

Due to the high heterogeneity among the studies, subgroup analysis by population was reported in Table 4. The highest prevalence of depression and anxiety in patients with TS in the adult population was estimated to be 47.9% (95% confidence interval: 27.6–68.9%) and 61.3% (95% confidence interval: 34.7–82.5%), respectively (Table 4).

**Table 4:** Subgroup analysis of the prevalence of depression and anxiety

Subgroups		Number studies	Point estimate	Lower limit	Upper limit	Z-value	*P*-value	*P*-value between	I^2^	Standard error	Variance	Tau
Depression	Adult	4	0.479	0.276	0.689	-0.186	0.852	0.141	96.99	0.907	0.823	0.849
	Children	2	0.191	0.078	0.397	-2.761	0.006		84.92	0.790	0.624	0.689
	Children and Adolescents	6	0.363	0.156	0.639	-0.971	0.331		96.98	1.728	2.988	1.373
Anxiety	Adult	4	0.613	0.347	0.825	0.824	0.410	0.260	98.03	1.171	1.401	1.082
	Children and Adolescents	5	0.448	0.353	0.547	-1.030	0.303		74.67	0.133	0.154	0.364

## Discussion

The present systematic review and meta-analysis was performed to determine the prevalence of depression and anxiety in patients with TS. Finally, after combining the data obtained from 12 articles entered the meta-analysis, the prevalence of depression and anxiety was 36.4% and 53.5% in these patients, respectively. The highest prevalence of depression and anxiety was reported by Gharatya et al. (75% and 80%, respectively) [20]. The lowest prevalence of depression was reported by Robertson et al. (8.7%) [24] and the lowest prevalence of anxiety was reported by Baglioni et al. (31.4%) [25]. The highest quality assessment score [8] based on JBI checklist was related to a 2017 study in Italy [23], which reported a 66.7% prevalence of depression and a 44.8% prevalence of anxiety in patients with TS.

To the best of our knowledge, no systematic review or meta-analysis has been performed to estimate the prevalence of depression and anxiety in patients with TS. According to a meta-analysis by Kisely et al., the prevalence of depression and anxiety was 12.5% ​​and 3.4% in the general population, respectively [28]. Also, Salari et al. reported the prevalence of anxiety and depression 29.6% and 33.7% in the general population during Covid-19 pandemic, respectively [29]. Comparison of the results of the above meta-analyzes with the present study showed that the prevalence of depression and anxiety in patients with TS was higher than the general population, which requires special attention of health officials and policy makers.

There is evidence of dopamine involvement in tic disorders, based on the fact that dopamine antagonist drugs such as haloperidol suppress tics and factors that increase central dopaminergic activity, such as Ritalin, exacerbate tics. The relationship between tics and dopamine is not a simple one and has not yet been fully known [30]. There is also evidence of dysfunction of the cerebral cortex circuits involved in motor functions. Studies using magnetic resonance imaging have shown natural asymmetry of the tail nuclei in those with these disorders [31]. Environmental and social factors also play a role in the development of this syndrome, such as smoking and high levels of stress during pregnancy, prematurity and low birth weight, psychiatric disorders, streptococcal infections and other psychological stresses. Therefore, therapeutic approaches to tic disorders can be divided into three main groups: medication, cognitive-behavioral therapy, and behavioral therapy [32].

The onset of Tourette syndrome is usually between 4 and 6 years old. The highest severity occurs between the ages of 10 and 12. So that in adolescence its intensity reduces. Many adults with Tourette syndrome experience reduced symptoms [4]. However, the results of subgroup analysis of the present study showed that the prevalence of depression and anxiety in patients with TS in the adult population was higher than other populations studied. Also, the results of meta-regression showed that by increasing mean age (9–31.5 years), the prevalence of depression and anxiety in patients with TS increased significantly. The reasons for the higher prevalence of depression and anxiety with age can be the influence of social environment, university and work, pubertal stress, substance abuse, negative thinking patterns, differences in the brain (adolescents' brain is structurally different from adults' brain), changes in the brain circuits of adolescents play a role in the risk-reward response, and increase stress levels. The adolescents with anxiety and depression have different neurotransmitters, including dopamine, serotonin, and norepinephrine in their brains affecting mood and behavior) and etc.

The high prevalence of depression and anxiety in patients with TS in different populations, especially in young people, indicates the need for investigation and follow-up for these disorders in these patients. Due to the complications and problems that depression and anxiety cause in these patients and its significant effect on various aspects of life, there is a need for special attention by the authorities. In order to reduce the prevalence of depression and anxiety, one should become aware of this issue, find the appropriate solution, implement these solutions, and follow up the results of the actions. This policy is effective when implemented at the individual, group and organizational levels.

Due to the small number of studies included in the meta-analysis in the systematic review and meta-analysis of the present study and most of the articles were presented in continental Europe, it was not possible to analyze subgroups according to the continent and social environment studied. Due to the influence of culture and social environment on the prevalence of depression and anxiety, it is suggested to conduct further studies with larger sample sizes in different parts of the world, continents and cultures to determine the prevalence of these disorders more accurately in different populations and cultures.

High heterogeneity between studies (90%>) led to perform a subgroup analysis according to the study population, which reduced a small amount of heterogeneity between studies, but still heterogeneity in all subgroups is high, which may be due to demographic information, sample size and study methodology. Other limitations included the lack of uniform reporting of articles, the same method, the random selection of some samples, the small sample size of some articles, the small number of studies in some subgroups for subgroup analysis and the lack of access to the full text of articles presented at the conference.

## Conclusion

The results of the present systematic review and meta-analysis showed that the prevalence of depression and anxiety in patients with TS was high. Therefore, it is suggested that health officials and policy makers design measures to prevent and control these disorders.

## Data Availability

Datasets are available through the corresponding author upon reasonable request.

## Abbreviations

TS: Tourette Syndrome
WoS: Web of Science
MeSH: Medical Subject Headings
PRISMA: Preferred Reporting Items for Systematic Reviews and Meta-Analysis
BCDI: Birleson Child Ikpression Invcntory
HDRS: Hamilton Depression Rating Scale
TBSA: Tyrer’s Brief Scale for Anxiety
BDI: Beck Depression Inventory
CDI: Child Depression Inventory
MASC: Multidimensional Anxiety Scale for Children
DSM: Diagnostic and Statistical Manual of Mental Disorders
